# Anatomy, Descriptive and Applied

**Published:** 1910-11

**Authors:** 


**Anatomy, Descriptive and Applied.** By **Henry Gray,** F.R.S., late Lecturer on Anatomy at St. George's Hospital, London. Eighteenth edition revised by **Edward Anthony Spitzka,** M.D., **Professor** of Anatomy in the Jefferson Medical College of Philadelphia. Imperial octavo, 1496 pages with 1208 engravings. Philadelphia and New York: Lea & Febiger, Publishers. (Cloth, $6.00; leather, $7.00 net prices.)

The eighteenth edition of Gray's Anatomy is now ready for distribution. For more than fifty years this work has held the leading place in its own subject and, measured by the number of students who have used it, is incomparably the best-known medical book in the English language. The arrangement in the new revision is along the old familiar lines adopted by its brilliant and versatile author when he laid so broadly and so well his conception of how anatomy should be studied. In this edition some illustrations not thought to be of prime importance have been omitted, others have been redrawn, and many new ones are added. The language of the text has in many places been simplified and rearranged with no loss of clearness, and with no increase in the size of the book. It will still claim first place among anatomical works for the student. J. A. R.

